# Evaluation of Silica-H_2_SO_4_ as an Efficient Heterogeneous Catalyst for the Synthesis of Chalcones

**DOI:** 10.3390/molecules180810081

**Published:** 2013-08-20

**Authors:** Aeysha Sultan, Abdul Rauf Raza, Muhammad Abbas, Khalid Mohammed Khan, Muhammad Nawaz Tahir, Nazamid Saari

**Affiliations:** 1 Ibn e Sina Block, Department of Chemistry, University of Sargodha, Sargodha 40100, Pakistan; E-Mails: blackhawk.aries@gmail.com (A.S.); abbas12396@yahoo.com (M.A.); 2 HEJ Research Institute of Chemistry, International Centre for Chemical & Biological Sciences, University of Karachi, Karachi 75270, Pakistan; E-Mail: khalid.khan@iccs.edu; 3 Ibn ul Haithum Block, Department of Physics, University of Sargodha, Sargodha 40100, Pakistan; E-Mail: dmntahir_uos@yahoo.com; 4 Department of Food Science, University Putra Malaysia, UPM 43400, Serdang, Malaysia; E-Mail: nazamid@upm.edu.my

**Keywords:** silica-H_2_SO_4_, solvent free conditions, chalcone, arylidene indanone, arylidene tetralone, Claisen-Schmidt condensation

## Abstract

We report an efficient silica-H_2_SO_4_ mediated synthesis of a variety of chalcones that afforded the targeted compounds in very good yield compared to base catalyzed solvent free conditions as well as acid or base catalyzed refluxing conditions.

## 1. Introduction

The generic term chalcones refer to compounds with a main 1,3-diphenylprop-2-enone core. Chemically chalcones are open chain flavonoids with two aromatic rings linked *via* a three carbon α,β-unsaturated enone system. These compounds are widely found in numerous species of plant, which are used as traditional folk medicines for treatment of a large number of diseases. Whether synthetic or isolated from plants, chalcones have been found to be associated with diverse biological applications such as antiinflammatory [1], antipyretic, antimutagenic [2], antioxidant [3], cytotoxic, antitumor [4] and a large list yet to be mentioned.

Owing to their diverse biological activities, many synthetic strategies toward these compounds have been developed that involve Claisen-Schmidt condensations of substituted acetophenones with aldehydes. Different reagents employed for the chalcone synthesis include aq. alcoholic alkali [5], dry HCl [6], anhydrous AlCl_3_ [7], POCl_3_ [8], aqueous Na_2_B_4_O_7_·10H_2_O [9], HClO_4_ [10], BF_3_ [11], Mg(OtBu)_2_ [12], graphite oxide [13,14] hydroxyapetite [15,16], phosphate derivatives [17], organo Cd compounds, SnCl_4_ and the use of animal bone meal (ABM) as a heterogeneous catalyst [18]. In addition to these Gupta and Boss *et al.,* in their separate studies synthesized chalcones under microwave irradiation in the presence of NaOH [19,20]. Seedhar *et al.*, carried out chalcone synthesis in polyethylene glycol (PEG) as an environment friendly solvent [21]. Boukhvalov *et al.* carried out a computational investigation of the potential role of graphene oxide as a heterogenous catalyst [22].

With increasing concerns about environmental pollution, synthetic strategies are been developed that involve the use of less or no solvent. Similarly the heterogeneous catalysis is preferred over homogenous catalysis because of the work-up, economical and environmental advantages of the former. Silica-H_2_SO_4_ (SSA) is a versatile, selective and a powerful catalyst that has been explored for various organic transformations, such as the synthesis of heterocyclic compounds [23,24,25,26,27], cross-aldol condensations [28], Michael additions [29], protection [30,31], deprotection [32] and oxidation reactions [33]. The major advantages of SSA include: ease of preparation, ease of removal from reaction mixtures, comparatively mild conditions as compared to H_2_SO_4_ as well as NaOH. Since it requires no use of solvent, therefore it is economical as well as environmentally friendly and most important thing is that it can be recycled.

In this article, we wish to report an efficient and versatile procedure for the synthesis of chalcones in the presence of SSA and a comparison of the results of our synthesis to different methods in order to evaluate the effectiveness of the SSA-mediated synthesis of chalcones.

## 2. Results and Discussion

For the preparation of chalcones, four different reagents/reaction conditions were chosen: refluxing conditions using MeOH as a solvent in the presence of stoichiometric amount of H_2_SO_4_ or NaOH, grinding the reactants with NaOH pallets under neat conditions (SF) and by heating the reactants with SSA in the absence of any solvent.

The SSA was prepared by two different reported methods. One method involves the addition of H_2_SO_4_ to a suspension of silica gel in Et_2_O, followed by the evaporation of the solvent under reduced pressure and heating the resulting silica gel at 120 °C for 3 h [34]. The other method involves the addition of silica gel to HSO_3_Cl along with subsequent trapping of HCl produced during the reaction [35]. The SSA obtained by both methods was similar in form, *i.e.*, a white solid, and showed similar results.

**Scheme 1:**
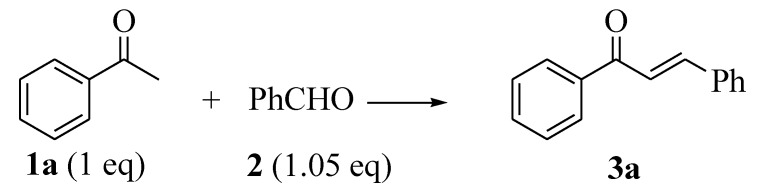
SSA-assisted synthesis of chalcone **3a**.

In order to determine the optimum amount of SSA required for a given transformation, the simplest chalcone **3a** (obtained by condensing PhAc with PhCHO in the presence of varying amounts of SSA from 0.005 to 0.1 g) was synthesized (Scheme 1).

It is observed that best results are obtained with 0.02 g of SSA. If less than 0.02 g of SSA was employed the yield of the product was low or the transformation was incomplete. An increase in amount of SSA resulted in a slight increase in yield, but decomposition of the product and difficult isolation of the product was observed upon increasing (≥0.05 g) the amount of SSA (Table 1).

**Table 1:** Determination of optimum amount of SSA for the preparation of chalcone **3a**.

Entry	SSA (g)	Solvent	Time (Temperature, °C)	%Yield ^¥^
1	0.005	MeOH	4 h (reflux)	*
2	0.01	CH_2_Cl_2_	6 h (reflux)	*
3	0.01	-	2 h (65)	28
4	0.02	-	8 h (rt)	-
5	0.02	-	1 h (65)	91
6	0.02	-	0.5 h (100)	#
7	0.05	-	0.5 h (65)	94
8	0.1	-	0.5 h (65)	^

* A number of spots were observed on TLC along with reactants; ^#^ the SSA became a black powder and reaction workup afforded a number of spots on TLC; ^^^ no product could be isolated and TLC of the reaction mixture indicated the formation of a number of compounds; ^¥^ All yields reported above are isolated yields.

In order to confirm the effectiveness of SSA three control experiments were performed, which include heating reactants with silica gel under solvent free conditions, using H_2_SO_4_ (without silica gel) in MeOH (at 65 °C) and by heating the aldehyde and ketone in the presence of silica gel and H_2_SO_4_ at 65 °C both in the presence and absence of methanol (used as a solvent). No product formation was observed when only silica gel was used. When the reactants were heated together with silica and H_2_SO_4_ in the absence of solvent, blackening of the contents of reaction flask was observed with no transformation occurred, even after 4 h. Heating the reactants with silica and H_2_SO_4_ in MeOH yielded 1,3-diphenylprop-2-enone (**3a**) in less than 10% yield after 5 h. Heating the reactants in H_2_SO_4_ using MeOH at 65 °C afforded the chalcone **3a** in 28% yield after 4 h; however, refluxing the methanolic solution of reactants with H_2_SO_4_ afforded chalcone **3a** in 38% after 4 h.

The catalyst is not only removed easily, but can be recycled. The catalyst was recovered by simple filtration after the addition of CH_2_Cl_2_ followed by partitioning between H_2_O and the organic layer. The residual catalyst was washed with acetone in order to extract any remaining product adsorbed on the catalyst surface, and it was then reactivated by placing in an oven for 30 min at 100 °C. The recovered catalyst was used three times for the synthesis of 1,3-diphenylprop-2-enone and almost the same yield was obtained as observed in the first run.

### 2.1. Synthesis of Open Chain Chalcones **3a**–**o**

**Scheme 2:**
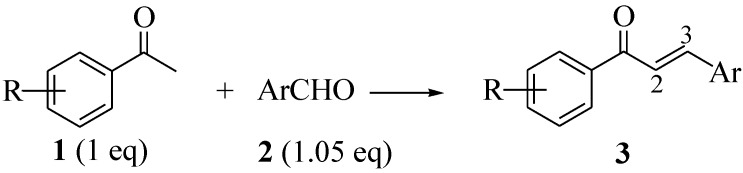
Synthesis of chalcones **3** under different reaction conditions.

When substituted PhAc **1** and ArCHO **2** were condensed in the presence of different reagents, the capricious yield of the products **3** depends upon the nature of reagent used. In general, the base- catalyzed reaction under refluxing conditions gave the lowest yields in almost all cases. The effect was more pronounced when either substrate (*i.e.*, **1** or **2**) contains –I and +R groups (such as OH, NMe_2_) or –I and –R groups (such as NO_2_). The acid catalyzed reaction also suffered the problem of low yields. The low yield with base-catalyzed refluxing conditions was attributed to the oxidation of aldehydes to their corresponding carboxylic acids *via* the Cannizarro reaction, which results in an overall decrease in the active concentration of aldehyde **2**. The oxidation of aldehydes to carboxylic acids was much pronounced with *para*-substituted **2**. The solvent free (SF) conditions led to quite a high yield of the product; however, the yields were quite low when either or both of the reactants contains –I and +R/–R groups. The yields of such substrates under SSA conditions are quite higher (Scheme 2. Table 2). The formation of the chalcones **3a**–**o** was confirmed by ^1^H-NMR that indicated the presence of *J_trans_* (14.9–17.4 Hz). The mass spectra were also in agreement with the formation of the targeted chalcones.

**Table 2:** Comparison of yield using different reagents and δ of olefinic protons in **3a**–**o**.

Entry	R	Ar	3	%Yield				^1^H-δ ^!^ (*J*^§^)	
				H_2_SO_4_^*^	NaOH ^#^	SF^^^	SSA ^¥^	H^2^	H^3^
**a**	H	Ph	**3a**	54	45	77	91	7.60 (16.4)	8.12 (16.4)
**b**	3′-OH	Ph	**3b**	25	51	82	95	7.58 (15.9)	7.98 (15.9)
**c**	3′-OH	2-furyl	**3c**	38	43	71	83	7.35 (16.8)	7.59 (16.8)
**d**	4′-OH	Ph	**3d**	48	24	73	88	7.43 (15.6)	7.81 (15.6)
**e**	4′-OH	2-furyl	**3e**	45	41	85	88	7.54 (17.1)	7.83 (17.4)
**f**	4′-OH	4-MeOPh	**3f**	58	32	89	92	7.43 (15.4)	7.79 (15.4)
**g**	4′-Me	Ph	**3g**	62	73	92	89	7.56 (17.4)	7.88 (17.4)
**h**	4′-Me	2-furyl	**3h**	54	79	86	94	7.55 (15.6)	7.88 (15.6)
**i**	4′-Me	4-Me_2_NPh	**3i**	<10	13	29	80	6.86 (14.9)	7.58 (14.9)
**j**	3′-NO_2_	Ph	**3j**	<10	-	35	83	7.62 (16.0)	8.02 (16.0)
**k**	3′-NO_2_	2-furyl	**3k**	15	-	40	87	7.50 (16.8)	7.77 (16.8)
**l**	3′-NO_2_	4-Me_2_NPh	**3l**	23	-	25	76	7.54 (15.8)	7.83 (15.8)
**m**	3′-NO_2_	4-MeOPh	**3m**	19	-	33	74	7.38 (16.0)	7.79 (16.0)
**n**	4′-Cl	Ph	**3n**	78	68	83	96	7.61 (16.6)	8.18 (16.5)
**o**	4′-Cl	2- MeOPh	**3o**	75	64	76	92	7.63 (16.1)	8.03 (16.1)

* 1.5 equivalent to **1**, 5 h reflux in MeOH; ^#^ 1.15 equivalents to **1**, 3 h reflux; ^^^SF (NaOH mediated solvent free) 3 equivalents of NaOH to **1**, grinding in neat conditions; ^¥^ SSA heating at 65 °C for 1.5 h under neat conditions; ^!^ chemical shifts are reported in ppm; ^§^ coupling constants are reported in Hz (both protons showed doublets in all cases).

### 2.2. Synthesis of Tetralone- and Indanone-Based Chalcones **5a**–**m**

**Scheme 3:**
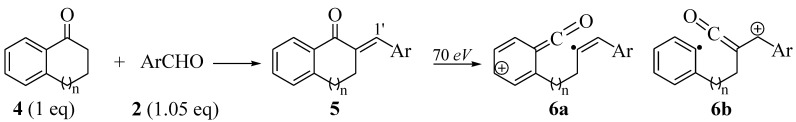
Synthesis of arylidene tetralone and arylidene indanones **5** under different conditions.

After the successful synthesis of various substituted chalcones **3a**–**o**, the effect of reagent on the yield of tetralone- and indanone-based chalcones was studied. For this purpose the tetralone and/or indanone was allowed to condense with various aldehydes in the presence of acid, base, solvent free conditions and SSA. The trends were almost similar as observed in case of **3a**–**o**. In most cases a molecular ion **6a** or **6b** was observed as a stable radical cation (Scheme 3, Table 3).

**Table 3:** Comparison of yield using different reagents, δ of H^1′^ and *m/z* of [M]^+·^ in **5a**–**m**.

Entry	n	Ar	5	%Yield				^1^H-δ ^!^ (H^1′^)	[6a]+ or [6b]+(% abundance)
				H_2_SO_4_ *	NaOH^#^	SF^^^	SSA ^¥^		
**a**	0	Ph	**5a**	35	41	83	87	7.93	220 (64)
**b**	0	2-furyl	**5b**	48	52	78	91	7.44	210 (100)
**c**	0	4-Me_2_NPh	**5c**	12	-	85	82	6.97	263 (100)
**d**	0	4-MeOPh	**5d**	73	35	82	97	8.13	250 (76)
**e**	0	3-MeOPh	**5e**	75	56	79	90	7.64	250 (100)
**f**	0	3-NO_2_Ph	**5f**	13	<10	53	72	8.53	265 (42)
**g**	0	3,4-(OMe)_2_Ph	**5g**	75	63	80	86	7.18	280 (100)
**h**	1	Ph	**5h**	66	54	76	80	6.98	234 (56)
**i**	1	2-furyl	**5i**	72	68	85	87	7.56	224 (100)
**j**	1	4-Me_2_NPh	**5j**	21	17	62	94	7.82	277 (52)
**k**	1	4-MeOPh	**5k**	76	71	85	89	6.69	264 (100)
**l**	1	3-NO_2_Ph	**5l**	20	19	71	85	8.27	279 (29)
**m**	1	3-ClPh	**5m**	78	42	79	87	6.81	268, 270 (38, 12)

* 1.5 equivalent to **1**, 5 h reflux in MeOH; ^#^ 1.01 equivalents to **1**, 3 h reflux; ^^^ SF (NaOH mediated solvent free) 3 equivalents of NaOH to **1**, grinding under neat conditions; ^¥^ SSA (0.02 g), heating at 65 °C for 1.5 h under neat condition; ^!^chemical shifts are reported in ppm.

**Figure 1:**
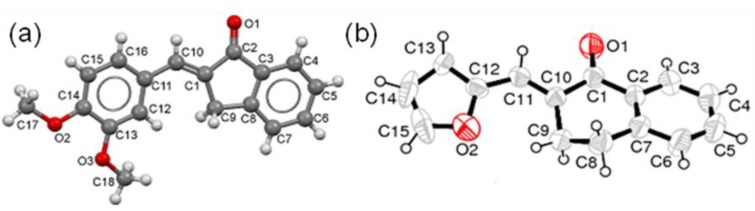
The ORTEP diagram of (**a**) **5g**. (**b**) **5i**.

The change in ring size of tetralone and indanone didn’t affect the yield of the product(s). The formation of arylidene indanone/tetralones was confirmed by ^1^H-NMR that indicated the presence of an olefinic proton that appeared as a singlet (6.69–7.82 ppm) in most of the cases depending upon the –I and –R/+R effect of the locants at **2** (Figure 3). The XRD of a couple of products (**5g** and **5i**, one from each case) confirmed the formation of a new C=C bond (1.337Å between C1 & C10 and 1.340Å between C10 & C11 respectively) (Figure 1) [36].

**Figure 2:**
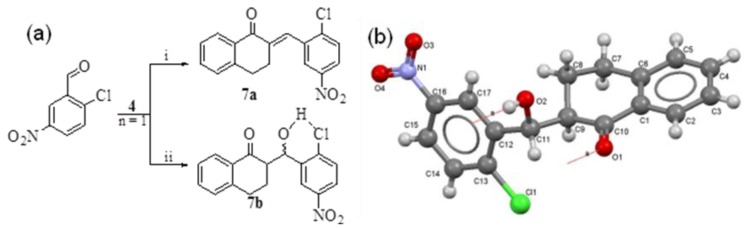
(**a**) Formation of **7a** and **7b** under different reaction conditions: **i**) NaOH reflux (**7a**, 43%), SF (**7a**, 71%); SSA (**7a**, 82%); **ii**) H_2_SO_4_ reflux (**7b**, 73%). (**b**) The ORTEP diagram of **7b**.

An aldol product **7b** was isolated as a major product in case of H_2_SO_4_-mediated condensation of **4** with 2-Cl-5-NO_2_PhCHO; whereas the base or SSA catalyzed reactions afforded the desired enone **7a**. Due to steric factors no *o*-substituted substrate was used in any previous case. The H-bonding, forming a six member ring, between Cl or carbonyl O and alcoholic H would probably be the reason of the failure of the dehydration in **7b** (Figure 2a). The XRD of **7b** showed a new C-O (1.418Å) and O-H (0.821Å) bond formation instead of C=C (Figure 2b) [37].

## 3. Experimental

The TLC was carried out on pre-coated silica gel (0.25 mm thick layer over Al sheet, Merck, Darmstadt, Germany) with fluorescent indicator. The spots were visualized under UV lamps (λ 365 and 254 nm) of 8 W power or KMnO_4_ dip and heating. The compounds were purified either on a glass column packed silica gel (0.6–0.2 mm, 60Å mesh size, Merck) or by crystallization. All solutions were concentrated under reduced pressure (25 mm of Hg) on a rotary evaporator (Laborota 4001, Heidolph, Germany) at 35–40 °C. Melting points were determined using a MF-8 (Gallenkamp, Burladingen, Germany) instrument and are reported uncorrected. The IR-spectra are recorded on Prestige 21 spectrophotometer (Shimadzu, Japan) as KBr discs. The LREIMS are carried out on a Fisons Autospec Mass Spectrometer (VG, New Jersey, USA). The ^1^H (300, 400 and 500 MHz) and ^13^C-NMR (75 MHz) are recorded on AM-300, 400 and 500 MHz instruments (Bruker, Massachusetts, USA) in CDCl_3_ using TMS as internal standard.

### 3.1. Preparation of SSA

Method A: The H_2_SO_4_ was added to a stirred suspension of silica gel in Et_2_O. After stirring for 1 h, the solvent was evaporated under reduced pressure. The resulting SSA was placed in an oven at 120 °C for 3 h, which afforded SSA as a white solid.

Method B: The silica gel was added to HSO_3_Cl along with subsequent trapping of HCl produced during the reaction. The suspension thus formed was stirred at room temperature for 3 h and the resultant product was dried in fume-hood to remove any trapped HCl produced during the reaction. The SSA obtained in this manner was white sand like solid.

### 3.2. Representative Procedure for H_2_SO_4_ Catalyzed Synthesis of Chalcones under Reflux

The PhAc (1 mL, 0.90 g, 7.53 mmol, 1 eq.) and PhCHO (0.84 g, 7.91 mmol, 1.05 eq.) were added to a stirred solution of H_2_SO_4_ (0.5 mL, 0.86 g, 8.66 mmol, 1.15 eq.) in MeOH (15 mL) and the resulting reaction mixture was refluxed for 3 h. After the completion of reaction, the solvent was evaporated under a stream of N_2_. The resulting reaction mixture was neutralized with 10% aq. NaHCO_3_ and partitioned between H_2_O (50 mL) and EtOAc (3 × 25 mL). The combined organic extract was dried over anhydrous Na_2_SO_4_, filtered and concentrated under reduced pressure to afford product as white amorphous solid. Crystallization from CH_2_Cl_2_ afforded product as colorless needles (0.89 g, 54%).

### 3.3. Representative Procedure for NaOH Catalyzed Synthesis of Chalcones under Reflux

The PhAc (1 mL, 0.90 g, 7.53 mmol, 1 eq.) and PhCHO (0.84 g, 7.91 mmol, 1.05 eq.) were added to a stirred solution of NaOH (0.35 g, 8.66 mmol, 1.15 eq.) in MeOH (15 mL) and the resulting reaction mixture was refluxed for 3 h. After the completion of reaction, the solvent was evaporated under a stream of N_2_. The resulting reaction mixture was acidified with dil. *aq*. HCl and partitioned between H_2_O (50 mL) and EtOAc (3 × 25 mL). The combined organic extract was dried overanhydrous Na_2_SO_4_, filtered and concentrated under reduced pressure to afford product as white amorphous solid. Crystallization from CH_2_Cl_2_ afforded product as colorless needles (0.74 g, 45%).

### 3.4. Representative Procedure for NaOH Catalyzed Synthesis of Chalcones under Solvent Free Conditions

The PhAc (1 mL, 0.90 g, 7.53 mmol, 1 eq) and PhCHO (0.84 g, 7.91 mmol, 1.05 eq) were ground together in a mortar and pestle in the presence of NaOH (0.30 g, 7.60 mmol, 1.01 eq) for 30 min. The reaction mixture was neutralized and extracted with Et_2_O (3 × 25 mL). The combined organic extract was dried over anhydrous Na_2_SO_4_, filtered and concentrated under reduced pressure to afford the enone as colourless solid (1.27 g, 77%).

### 3.5. Representative Procedure for the SSA Catalysed Synthesis of Chalcones

The SSA (0.02 g) was added to a well stirred suspension of PhAc (1 mL, 0.90 g, 7.53 mmol, 1 eq.) and PhCHO (0.84 g, 7.91 mmol, 1.05 eq.) and the resulting mixture was heated at 65 °C for 1.5 h. The reaction mixture was cooled to room temperature and partitioned between brine (25 mL) and CH_2_Cl_2_ (3 × 15 mL) and solid SSA was filtered off. The SSA was washed with acetone (25 mL) to ensure desorption of product on SSA surface. The combined organic extract was washed with brine (3 × 25 mL) and the organic extract was dried over anhydrous Na_2_SO_4_, filtered and concentrated under reduced pressure to afford the chalcone as colorless solid (1.48 g, 91%).

*1,3-Diphenylprop-2-enone* (**3a**): R*_f_*: 0.58 (EtOAc/*n*-hexane, 1:3); M.p.: 57 °C (Lit. 56–57 °C)[38]; IR (KBr): ύ_max_ (cm^–1^) 2930 (=C-H), 1679 (C=O); ^1^H-NMR (400 MHz, CHCl_3_, δ in ppm): 7.18–7.32 (5H, m, H^2′^-H^4^^′^), 7.46-7.55 (3H, m, H^3′′^, H^4′′^), 7.60 (1H, d, *J* = 16.4 Hz, H^2^), 7.89 (2H, d, *J* = 7.8 Hz, H^2′′^), 8.12 (1H, d, *J* = 16.4 Hz, H^3^); ^13^C-NMR (75 MHz, CDCl_3_, δ in ppm): 123.3 (d, C^2^), 125.4, 126.9, 128.4, 129.2 (2 ×, d, C^2^^′^, C^3^^′^, C^2^^′′^, C^3^^′′^), 127.1, 133.8 (d, C^4^^′^, C^4^^′′^), 134.2, 135.5 (s, C^1^^′^, C^1^^′′^), 144.1 (d, C^3^), 187.5 (s, C^1^); EI-MS (*m/z*, amu): 208 [M]^+^ (54%), 131 [M − Ph]^+^ (100%), 105 [PhCO]^+^ (98%).

*3-(4*′*-Hydroxyphenyl)-1-phenylpropenone* (**3d**): R*_f_*: 0.48 (EtOAc/ *n*-hexane, 3:1); M.p.: 44 °C (Lit. 35–45 °C)[38]; IR (KBr): ύ_max_ (cm^–1^) 3320 (O-H, bs), 2885 (=C-H), 1669 (C=O); ^1^H-NMR (400 MHz, CHCl_3_, δ in ppm): 6.87 (2H, d, *J* = 7.6 Hz, H^3′^), 7.14–7.28 (5H, m, Ph-H), 7.43 (1H, d, *J* = 15.6 Hz, H^2^), 7.65 (2H, d, *J* = 7.5 Hz, H^2′^), 7.81 (1H, d, *J* = 15.6 Hz, H^3^), 10.10 (1H, bs, OH); ^13^C-NMR (75 MHz, CDCl_3_, δ in ppm): 112.8 (2×, d, C^3^^′^), 121.0 (d, C^2^), 126.2, 128.1, 129.9 (2 ×, d, C^2^^′^, C^2^^′′^, C^3^^′′^), 129.4 (s, C^1^^′′^), 128.3 (d, C^4^^′′^), 132.8 (s, C^1^^′^), 140.8 (d, C^3^), 158.3 (s, C^4^^′^), 187.6 (s, C^1^); EI-MS (*m/z*, amu): 224 [M]^+^ (54%), 131 [PhCH=CHCO]^+^ (100%, A), 121 [M − A]^+^ (94%).

*3-(Furan-2*′′*-yl)-1-p-tolylpropenone* (**3h**): R*_f_*: 0.53 (EtOAc/ *n*-hexane, 1:3); M.p.: 64–67 °C (Lit. 62–64 °C)[39]; IR (KBr): ύ_max_ (cm^–1^) 2898 (=C-H), 1672 (C=O); ^1^H-NMR (400 MHz, CHCl_3_, δ in ppm): 2.27 (3H, s, Me), 6.57 (1H, dd, *J* = 3.2, 1.8 Hz, H^4′′^), 6.78 (1H, d, *J* = 3.2 Hz, H^3′′^), 7.22 (2H, d, *J* = 7.5 Hz, H^3′^), 7.55 (1H, d, *J* = 15.6 Hz, H^2^), 7.67 (2H, d, *J* = 7.5 Hz, H^2′^), 7.82 (1H, d, *J* = 1.8 Hz, H^5′′^), 7.88 (1H, d, *J* = 15.6 Hz, H^3^); ^13^C-NMR (75 MHz, CDCl_3_, δ in ppm): 19.6 (q, CH_3_), 110.4, 111.9, 128.3, 129.2, 129.4, 129.6, 129.9 (d, C^2^, C^2^^′^, C^3^^′^, C^5^^′^, C^6^^′^, C^3^^′′^, C^4^^′′^), 134.1 (s, C^1^^′^/C^4^^′^), 141.8 (d, C^3^^′^/C^5^^′′^), 142.9 (s, C^1^^′^/C^4^^′^), 143.5 (d, C^3^^′^/C^5^^′^), 154.8 (s, C^1^^′′^), 189.3 (s, C^1^); EI-MS (*m/z*, amu): 212 [M]^+^ (79%), 121 [M − C_6_H_4_Me]^+^ (94%), 119 [MeC_6_H_4_CO]^+^ (100%).

*3-(4*′′*-Dimethylaminophenyl)-1-(3*′*-nitrophenyl)propenone* (**3l**): R*_f_*: 0.46 (EtOAc/ *n*-hexane, 1:1); M.p.: 107–111 °C (Lit. 110 °C)[40]; IR (KBr): ύ_max_ (cm^–1^) 2999 (=C-H), 1663 (C=O), 1545 (N=O); ^1^H-NMR (400 MHz, CHCl_3_, δ in ppm): 2.94 (6H, s, N(CH_3_)_2_), 6.54 (2H, d, *J* = 7.8 Hz, H^3′′^), 7.18 (2H, d, *J* = 7.8 Hz, H^2′′^), 7.54 (1H, d, *J* = 15.8 Hz, H^2^), 7.68 (1H, t, *J* = 6.8 Hz, H^5c^), 7.83 (1H, d, *J* = 15.8 Hz, H^3^), 7.96 (1H, dd, *J* = 6.8, 2.4 Hz, H^6′^), 8.31 (1H, d, *J* = 6.8 Hz, H^4′^), 8.66 (1H, t, *J* = 2.4 Hz, H^2′^); ^13^C-NMR (75 MHz, CDCl_3_, δ in ppm): 42.7, 42.9 (q, N-CH_3_), 112.9 (2×, d, C^3^^′′^), 120.2 (d, C^2^), 122.4 (s, C^1^^′′^), 126.7 (2×, d, C^2^^′′^), 129.8, 130.4, 130.8, 133.6 (d, C^2^^′^, C^4^^′^-C^6^^′^), 135.1 (s, C^1^^′^), 140.2 (s, C^4^^′′^), 144.4 (d, C^3^), 149.0 (s, C^3^^′^), 188.2 (s, C^1^); EI-MS (*m/z*, amu): 296 [M]^+^ (18%), 174 [M − C_6_H_4_NO_2_]^+^ (100%, A), 150 [NO_2_C_6_H_4_CO]^+^ (23%), 146 [A − CO]^+^ (100%). 

*1-(4*′*-Chlorophenyl)-3-(4*′′*-methoxyphenyl)propenone* (**3o**): R*_f_*: 0.57 (EtOAc/*n*-hexane, 1:1); M.p.: 67–68 °C (Lit. 68–70 °C)[41]; IR (KBr): ύ_max_ (cm^–1^) 3007 (=C-H), 1658 (C=O), 766 (C-Cl); ^1^H-NMR (400 MHz, CHCl_3_, δ in ppm): 3.73 (3H, s, OMe), 6.65 (2H, d, *J* = 7.8 Hz, H^3′′^), 6.98 (2H, d, *J* = 7.8 Hz, H^3′^), 7.41 (2H, d, *J* = 7.5 Hz, H^2′′^), 7.63 (1H, d, *J* = 16.1 Hz, H^2^), 7.67 (2H, d, *J* = 7.5 Hz, H^2′^), 8.03 (1H, d, *J* = 16.1 Hz, H^3^); ^13^C-NMR (75 MHz, CDCl_3_, δ in ppm): 63.2 (q, OCH_3_), 114.3 (2×, d, C^3^^′′^), 119.0 (s, C^2^), 127.3 (2×, d, C^2^^′^), 128.8 (2×, d, C^2^^′′^), 129.7 (2×, d, C^3′^), 130.2 (s, C^1′′^), 136.7 (s, C^4′^), 138.8 (s, C^1′^), 145.1 (d, C^3^), 161.0 (s, C^4′′^), 189.9 (s, C^1^); EI-MS (*m/z*, amu): 272, 274 [M]^+^ (56, 17%), 161 [M − C_6_H_4_Cl]^+^ (100%).

*2-(Furan-2*′′*-yl)methyleneindan-1-one* (**5b**): R*_f_*: 0.55 (EtOAc/ *n*-hexane, 1:3); M.p.: 116 °C (Lit. 118–119 °C)[42]; IR (KBr): ύ_max_ (cm^–1^) 2991 (=C-H), 1616 (C=O); ^1^H-NMR (CDCl_3_, 400 MHz, δ in ppm): 4.04 (2H, s, H^3^), 6.53–6.54 (1H, m, H^4^^′′^), 6.75 (1H, d, *J* = 3.2 Hz, H^3^^′′^), 7.41 (1H, t, *J* = 7.2 Hz, H^6^), 7.44 (1H, s, H^1^^′^), 7.53 (1H, d, *J* = 7.2, H^4^), 7.57-7.61 (2H, m, H^5^, H^5^^′′^), 7.87 (1H, d, *J* = 7.2 Hz, H^7^); ^13^C-NMR (75 MHz, CDCl_3_, δ in ppm): 20.9 (t, C^3^), 110.4, 112.3 (d, C^3^^′′^, C^4^^′′^), 125.1, 127.8, 128.2, 129.9 (d, C^4^-C^7^), 136.1 (d, C^1^^′^), 136.9, 137.5, 138.3 (s, C^2^, C^3a^, C^7a^), 142.8 (d, C^5^^′′^), 154.1 (s, C^2^^′′^), 188.7 (s, C^1^); EI-MS (*m/z*, amu): 210 [M]^+^ (100%), 182 [M − CO]^+^ (18%, A), 181 [A − H]^+^ (84%).

*2-(4*′′*-Dimethylaminobenzylidene)indan-1-one* (**5c**): Bright yellow solid; R*_f_*: 0.52 (EtOAc/*n*-hexane, 1:1); M.p.: 167 °C(Lit. 168 °C)[43]; IR (KBr): ύ_max_ (cm^–1^) 2933 (=C-H), 1656 (C=O); ^1^H-NMR (CDCl_3_, 400 MHz, δ in ppm): 3.07 (6H, s, NMe_2_), 4.00 (2H, s, H^3^), 6.97 (1H, s, H^1^^′^), 7.41 (1H, d, *J* = 6.4 Hz, H^4^), 7.54–7.63 (6H, m, H^5^, H^6^, H^2^^′′^, H^3^^′′^), 7.88 (1H, d, *J* = 6.8 Hz, H^7^); ^13^C-NMR (75 MHz, CDCl_3_, δ in ppm): 24.5 (t, C^3^), 43.2, 43.4 (q, N-CH_3_), 112.8 (2×, d, C^3^^′′^), 123.8 (s, C1^′′^), 125.2 (2×, d, C^2^^′′^), 126.7, 128.5, 129.4, 130.2 (d, C^4^-C^7^), 133.9 (d, C^1^^′^), 134.0, 137.2, 137.8 (s, C^2^, C^3a^, C^7a^), 143.5 (d, C^4^^′′^), 186.9 (s, C^1^); EI-MS (*m/z*, amu): 263 [M]^+^ (100%), 235 [M − CO]^+^ (43%, A), 234 [A − H]^+^ (71%).

*2-(3*′′*-Methoxybenzylidene)indan-1-one* (**5e**): Colorless solid; R*_f_*: 0.58 (EtOAc/ *n*-hexane, 1:3); M.p.: 135 °C (Lit. 138 °C)[44]; IR (KBr): ύ_max_ (cm^–1^) 2948 (=C-H), 1679 (C=O); ^1^H-NMR (CDCl_3_, 300 MHz, δ in ppm): 3.86 (3H, s, OCH_3_), 4.04 (2H, s, H^3^), 6.94 (1H, dd, *J* = 8.1, 1.8 Hz, H^6^^′′^), 7.18 (1H, bs, H^2^^′′^), 7.26 (1H, d, *J* = 8.0 Hz, H^4^^′′^), 7.37 (1H, t, *J* = 7.8 Hz, H^6^), 7.41 (1H, t, *J* = 7.2 Hz, H^5^^′′^), 7.54 (1H, d, *J* = 7.2 Hz, H^4^), 7.58–7.63 (2H, m, H^5^, H^1^^′^), 7.90 (1H, d, *J* = 7.5 Hz, H^7^); ^13^C-NMR (75 MHz, CDCl_3_, δ in ppm): 25.2 (t, C^3^), 56.8 (q, O-CH_3_), 110.5, 112.4, 117.5 (d, C^2^^′′^,C^4^^′′^, C^6^^′′^), 125.8, 126.5, 127.5, 128.9, 130.0 (d, C^4^-C^7^, C^5^^′′^), 135.4 (d, C^1^^′^), 135.8, 136.2, 137.9, 138.5 (s, C^2^, C^3a^, C^7a^, C^1^^′′^), 159.9 (s, C^3^^′′^), 187.9 (s, C^1^); EI-MS (*m/z*, amu): 250 [M]^+^ (100%), 249 [M − H]^+^ (56%).

*2-(3*′′*-Nitrobenzylidene)indan-1-one* (**5f**): Pale yellow solid; R*_f_*: 0.54 (EtOAc/ *n*-hexane, 3:1); M.p.: 119–121 °C; IR (KBr): ύ_max_ (cm^–1^) 2987 (=C-H), 1679 (C=O), 1565 (N=O); ^1^H-NMR (CDCl_3_, 500 MHz, δ in ppm): 4.11 (2H, s, H^3^), 7.45 (1H, t, *J* = 7.5 Hz, H^6^), 7.60 (1H, d, *J* = 7.5 Hz, H^4^), 7.64 (2H, t, *J* = 8.0 Hz, H^5^, H^5^^′′^), 7.68 (1H, dd, *J* = 2.0, 2.0 Hz, H^2^^′′^), 7.92 (1H, d, *J* = 7.5 Hz, H^4^^′′^), 7.93 (1H, d, *J* = 7.5 Hz, H^6^^′′^/H^7^), 8.24 (1H, dd, *J* = 8.0, 1.5 Hz, H^6′′^/H^7^), 8.53 (1H, s, H^1^^′^); ^13^C-NMR (75 MHz, CDCl_3_, δ in ppm): 24.8 (t, C^3^), 122.2, 124.0, 125.8, 126.5, 127.5, 128.9, 129.7, 130.0 (d, C^4^–C^7^, C^2^^′′^, C^4^^′′^–C^5^^′′^), 135.9 (d, C^1^^′^), 136.0, 136.2, 136.5, 137.4 (s, C^2^, C^3a^, C^7a^, C^1^^′′^), 147.7 (s, C^3^^′′^), 188.6 (s, C^1^); EI-MS (*m/z*, amu): 265 [M]^+^ (42%), 219 [M − NO_2_]^+^ (35%, A), 218 [A − H]^+^ (53%).

*2-(3*′′*,4*′′*-Dimethoxybenzylidene)indan-1-one* (**5g**): off-white solid; R*_f_*: 0.58 (EtOAc/*n*-hexane, 1:3); M.p.: 182 °C (Lit. 183-185 °C)[45]; IR (KBr): ύ_max_ (cm^–1^) 3018 (=C-H), 1652 (C=O); ^1^H-NMR (CDCl_3_, 400 MHz, δ in ppm): 3.93 (3H, s, OMe), 3.95 (3H, s, OMe), 4.02 (2H, s, H^3^), 6.95 (1H, d, *J* = 8.4 Hz, H^6^^′′^), 7.18 (1H, s, H^1^^′^), 7.30 (1H, d, *J* = 7.2 Hz, H^5^^′′^), 7.42 (1H, t, *J* = 7.2 Hz, H^6^), 7.54–7.62 (3H, m, H^4^, H^5^, H^2^^′′^), 7.90 (1H, d, *J* = 8.0 Hz, H^7^); ^13^C-NMR (75 MHz, CDCl_3_, δ in ppm): 23.8 (t, C^3^), 58.9, 61.4 (q, OCH_3_), 111.6, 113.3, 118.4 (d, C^2′′^, C^5′′^, C^6′′^), 125.8 (s, C^1′′^), 127.1, 128.9, 129.3, 132.3, 134.9 (d, C^4^-C^8^, C^1′^), 132.4, 136.6, 137.2 (s, C^2^, C^3a^, C^7a^), 146.8, 147.1 (s, C^3′′^, C^4′′^), 187.1 (s, C^1^); EI-MS (*m/z*, amu): 280 [M]^+^ (100%), 279 [M − H]^+^ (38%), 249 [M − OMe]^+^ (41%).

*2-Benzylidene-3,4-dihydro-2H-naphthalen-1-one* (**5h**): Pale yellow solid; R*_f_*: 0.64 (EtOAc/*n*-hexane, 1:3); M.p.: 96 °C (Lit. 96 °C)[46]; IR (KBr): ύ_max_ (cm^–1^) 3016 (=C-H), 1656 (C=O); ^1^H-NMR (500 MHz, CDCl_3_, δ in ppm): 2.99 (1H, t, *J* = 7.2 Hz, H^3^), 3.13 (2H, t, *J* = 7.2, H^4^), 6.98 (1H, bs, H^1^^′^), 7.21–7.66 (8H, m, H^5^-H^7^, H^2^^′′^-H^4^^′′^), 7.85 (1H, d, *J* = 7.6 Hz, H^8^); ^13^C-NMR (75 MHz, CDCl_3_, δ in ppm): 27.2, 28.9 (t, C^3^, C^4^), 127.1, 128.3 (2×, d, C^2^^′′^, C^3^^′′^), 128.5, 128.6, 129.5 (d, C^5^, C^7^, C^4^^′′^), 131.8, 132.5, 136.0 (d, C^6^, C^8^, C^1^^′^), 136.7 (3×, s, C^8a^, C^2^, C^1^^′′^), 144.2 (s, C^4a^), 188.6 (s, C^1^); EI-MS (*m/z*, amu): 234 [M]^+^ (56%), 206 [M − CO]^+^ (23%).

*2-(Furan-2*′′*-yl)methylene-3,4-dihydro-2H-naphthalen-1-one* (**5i**): Colourless solid; R*_f_*: 0.54 (EtOAc/*n*-hexane, 1:3); M.p.: 129–131 °C; IR (KBr): ύ_max_ (cm^–1^) 2965 (=C-H), 1621 (C=O); ^1^H-NMR (CDCl_3_, 300 MHz, δ in ppm): 3.01 (2H, t, *J* = 6.6 Hz, H^4^), 3.33 (2H, ddd, *J* = 5.1, 5.1, 1.8 Hz, H^3^), 6.53 (1H, dd, *J* = 3.3, 1.8 Hz, H^4^^′′^), 6.71 (1H, d, *J* = 3.3 Hz, H^3^^′′^), 7.27 (1H, d, *J* = 7.5 Hz, H^5^), 7.38 (1H, t, *J* = 7.5 Hz, H^7^), 7.48 (1H, ddd, *J* = 7.5, 7.5, 1.5 Hz, H^6^), 7.56 (1H, s, H^1^^′^), 7.60 (1H, d, *J* = 1.5 Hz, H^5^^′′^), 8.11 (1H, dd, *J* = 7.5, 1.2 Hz, H^8^); ^13^C-NMR (75 MHz, CDCl_3_, δ in ppm): 26.7, 28.4 (t, C^3^, C^4^), 112.2 (d, C^4′′^), 116.6 (s, C^3′′^), 122.8, 127.0 (d, C^5^, C^7^), 128.1 (2×, d, C^6^, C^8^), 131.9 (s, C^4a^/C^8a^), 133.1 (d, C^1′^), 133.6 (s, C^4a^/C^8a^), 143.5 (s, C^2^), 144.4 (d, C^5′′^), 152.5 (s, C^2′′^), 187.4 (s, C^1^); EI-MS (*m/z*, amu): 224 [M]^+^ (100%), 223 [M − H]^+^ (42%), 196 [M − CO]^+^ (26%).

*2-(4*′′*-Dimethylaminobenzylidene)-3,4-dihydro-2H-naphthalen-1-one* (**5j**): Bright yellow solid; R*_f_*: 0.54 (EtOAc/*n*-hexane, 1:3); M.p.: 35 °C (Lit. 35 °C)[46]; IR (KBr): ύ_max_ (cm^–1^) 2889 (=C-H), 1665 (C=O); ^1^H-NMR (CDCl_3_, 400 MHz, δ in ppm): 2.93 (4H, m, H^3^, H^4^), 3.11 [6H, s, N(CH3)2], 7.07–7.47 (7H, m, H^5-7^, H^2′′^, H^3′′^), 7.82 (1H, s, H^1′^), 8.09 (1H, d, *J* = 7.2 Hz, H^8^); ^13^C-NMR (75 MHz, CDCl_3_, δ in ppm): 27.8, 28.7 (t, C^3^, C^4^), 40.1 [2×, q, N(CH3)2], 111.6 (s, C^1′′^), 123.6 (2×, d, C^3′′^), 127.3, (2×, d, C^2′′^), 126.8, 127.8, 128.0, 131.0, 132.1 (d, C^5^, C^6^, C^7^, C^8^, C^1′^), 132.7, 134.5, 142.9 (s, C^2^, C^8a^, C^4a^), 150.6 (s, C^4′′^), 187.8 (s, C^1^); EI-MS (*m/z*, amu): 277 [M]^+^ (53%), 276 [M − H]^+^ (33%), 249 [M − CO]^+^ (8%).

*2-(4*′′*-Methoxybenzylidene)-3,4-dihydro-2H-naphthalen-1-one* (**5k**): Yellow solid; R*_f_*: 0.54 (EtOAc/*n*-hexane, 1:3); M.p. 92 °C (Lit. 92 °C)[46]; IR (KBr): ύ_max_ (cm^–1^) 2949 (=C-H), 1654 (C=O); ^1^H-NMR (CDCl_3_, 400 MHz, δ in ppm): 2.94 (2H, t, *J* = 7.2 Hz, H^3^), 3.10 (2H, t, *J* = 7.2 Hz, H^4^), 3.75 (3H, s, OMe), 6.69 (1H, s, H^1′^), 6.82 (2H, d, *J* = 6.8 Hz, H^3′′^), 7.15 (2H, d, *J* = 6.8 Hz, H^2′′^), 7.24–7.35 (2H, m, H^5^, H^7^), 7.44 (1H, ddd, *J* = 7.2, 7.2, 1.8 Hz, H^6^), 7.90 (1H, dd, *J* = 7.2, 1.8 Hz, H^8^); ^13^C-NMR (75 MHz, CDCl_3_, δ in ppm): 27.8, 28.7 (t, C^3^, C^4^), 66.1 (q, OCH_3_), 111.6 (s, C^1′′^), 121.8 (2×, d, C^3′′^), 127.9 (2×, d, C^2′′^), 125.4, 127.2, 128.5, 131.0, 132.7, (d, C^5^, C^6^, C^7^, C^8^, C^1′^), 134.0 (s, C^2^), 142.9, 147.8 (s, C^4a^, C^8a^), 187.4 (s, C^1^); EI-MS (*m/z*, amu): 264 [M]^+^ (100%), 236 [M − CO]^+^ (88%).

*2-(3*′′*-Chlorobenzylidene)-3,4-dihydro-2H-naphthalen-1-one* (**5m**): Dull-brown solid; R*_f_*: 0.56 (EtOAc/*n*-hexane, 1:3); M.p.: 71–74 °C (Lit. 72 °C)[46]; IR (KBr): ύ_max_ (cm^–1^) 2948 (=C-H), 1662 (C=O), 785 (C-Cl); ^1^H-NMR (CDCl_3_, 300 MHz, δ in ppm): 2.98 (2H, t, *J* = 7.2 Hz, H^3^), 3.08 (2H, t, *J* = 7.2 Hz, H^4^), 6.81 (1H, s, H^1′^), 7.18–7.24 (3H, m, H^2′′^, H^4′′^, H^5′′^), 7.33-7.47 (4H, m, H^5^-H^7^, H^6′′^), 7.86 (1H, d, *J* = 7.2 Hz, H^8^); ^13^C-NMR (75 MHz, CDCl_3_, δ in ppm): 27.2, 28.7 (t, C^3^, C^4^), 123.1, 124.2, 127.3, 128.4, 129.6, 133.1, 133.6, 133.8 (d, C^5^, C^6^, C^7^, C^8^, C^2′′^, C^4′′^, C^5′′^, C^6′′^), 134.4, 135.8, 137.5, 137.8, 148.3 (s, C^2^, C^1′′^, C^3′′^, C^4a^, C^8a^), 187.3 (s, C^1^); EI-MS (*m/z*, amu): 268, 270 [M]^+^ (38, 12%), 240, 242 [M − CO]^+^ (18, 7).

(*E*)-*2-(2*′′*-Chloro-5*′′*-nitrobenzylidene)-3,4-dihydronaphthalen-1(2H)-one* (**7a**): Off-white solid; R*_f_*: 0.57 (EtOAc/*n*-hexane, 1:3); M.p.: 97 °C (Lit. 97 °C)[47]; IR (KBr): ύ_max_ (cm^–1^) 2968 (=C-H), 1681 (C=O), 1519 (N=O), 738 (C-Cl); ^1^H-NMR (300 MHz, CDCl_3_, δ in ppm): 2.99 (4H, s, H^3^, H^4^), 7.24 (1H, m, H^5^), 7.38 (1H, t, *J* = 7.2 Hz, H^7^), 7.51 (1H, t, *J* = 7.2 Hz, H^6^), 7.62 (1H, d, *J* = 8.8 Hz, H^8^), 7.82 (1H, s, H^1′^), 8.14-8.19 (3H, m, H^3′′^, H^4′′^& H^6′′^); ^13^C-NMR (75 MHz, CDCl_3_, δ in ppm): 24.2, 28.4 (t, C^3^, C^4^), 123.5, 123.8, 126.4, 128.5, 128.9, 129.4, 132.5, 133.6 (d, C^5^-C^8^, C^1^^′^, C^3^^′′^, C^4^^′′^, C^6^^′′^), 136.4, 136.8, 137.3, 137.5, 139.4, 143.4 (s, C_2_, C^4a^, C^8a^, C^1^^′′^, C^2^^′′^, C^4^^′′^), 187.8 (s, C^1^); EI-MS (*m/z*, amu): 315, 317 [M]^+^ (21, 6%), 280 [M − Cl]^+^ (81%).

## 4. Conclusions

The higher yields (72%–97%) of chalcones are obtained by SSA-mediated coupling over other reported strategies. Furthermore, the slightly lower yields of the hydroxyl substituted chalcones from solvent free NaOH mediated condensation makes SSA the method of choice for the synthesis of chalcones.

## References

[1] Hsieh H.K., Lee T.H., Wang J.P., Wang J.J., Kin C.N. (1998). Synthesis and anti-inflammatory effects of chalcones and related compounds. Pharm. Res..

[2] Torigoo T., Arisawa M., Iloch S., Fujiu M., Mayuyama H.B. (1983). Antimutagenic chalcones: Antagonizing the mutagenicity of benzo(a)pyrene in *Salmonella typhymurium*. Biochem. Biophys. Res. Commun..

[3] Haraguchi H., Ishikawa H., Mizutani K., Tamura Y., Kinoshira T. (1998). Antioxidant and superoxide scavenging activities of retrochalcones in *Glycyrrhiza inflate*. Bioorg. Med. Chem..

[4] De Vincenzo R., Ferlini C., Distefeno M., Gaggini C., Riva A., Bombardelli E., Morazzini P., Belluti F., Ranelletti F.O., Mancuso S. (2000). *In vitro* evaluation of newly developed chalcone analogues in human cancer cells. Cancer Chem. Pharmacol..

[5] Geissman T.A., Clinton R.O.  (1946). Flavanones and related compounds. I. The preparation of polyhydroxychalcones and flavanones. J. Am. Chem. Soc..

[6] Russel A., Todd S. (1939). The constitution of natural tannins. VI.^1^ Coloring matters derived from 2,5-dihydroxyacetophenone. J. Am. Chem. Soc..

[7] Zwaagstra M.E., Timmerman H., Tamura M., Tohma T., Wada Y., Onogi K., Zhang M. (1997). Synthesis and structure activity relationships of carboxylated chalcones:  A novel series of *CysLT_1_* (LTD_4_) receptor antagonists. J. Med. Chem..

[8] Davey W., Tivey D.J. (1958). Chalcones and related compounds. Part IV. Addition of hydrogen cyanide to chalcones. J. Chem. Soc..

[9] Jadhav G.V., Kulkarni V.G. (1951). Borax as a new condensing agent for the synthesis of chalkones. Curr. Sci..

[10] Matsushima R., Murakami T. (2000). Photoreactions of 3-(2-Hydroxyphenyl)-1-substituted phenyl-2-propen-1-ones (Substituted 2-Hydroxychalcones) in organic solvents in the presence and absence of acid. Bull. Chem. Soc..

[11] Breslow D.S., Hauser C.R. (1940). Condensations. ^1^ XI. Condensations of certain active hydrogen compounds effected by BF_3_ and AlCl_3_. J. Am. Chem. Soc..

[12] Guthrie J.L., Rabjohm N. (1957). Some reactions effected by means of bromomagnesium t-alkoxides. J. Org. Chem..

[13] Jia H.P., Dreyer D.R., Bielawski C.W. (2011). Graphite oxide as an auto-tandem oxidation-hydration-aldol coupling catalyst. Adv. Synth. Catal..

[14] Dreyer D.R., Bielawski C.W. (2011). Carbocatalysis: Heterogeneous carbons finding utility in synthetic chemistry. Chem. Sci..

[15] Solhy A., Tahir R., Sebti S., Skouta R., Bousmina M., Zahouily M., Larzek M. (2010). Efficient synthesis of chalcone derivatives catalyzed by re-usable hydroxyapatite. Appl. Catal. A.

[16] Sebti S., Solhy A., Tahir R., Smahi A. (2002). Modified hydroxyapatite with sodium nitrate: an efficient new solid catalyst for the Claisen-Schmidt condensation. Appl. Catal. A.

[17] Sebti S., Solhy A., Tahir R., Abdelatif S., Boulaajaj S., Mayoral J.A., García J.I., Fraile J.M., Kossir A., Oumimoun H. (2003). Application of natural phosphate modified with sodium nitrate in the synthesis of chalcones: a soft and clean method. J. Catal..

[18] Riadi Y., Abrouki Y., Mamouni R., El Haddad M., Routier S., Guillaumet G., Lazar S. (2012). New eco-friendly animal bone meal catalysts for preparation of chalcones and aza-Michael adducts. Chem. Cent. J..

[19] Gupta R., Paul S., Gupta A. (1995). Improved microwave-induced synthesis of chalcones and related enones. Ind. J. Chem..

[20] Boss A.K., Manhas M.S., Gosh M.S. (1991). Microwave-induced organic reaction enhancement chemistry. 2. Simplified techniques. J. Org. Chem..

[21] Seedhar N.Y., Jayapal M.R., Prasad K.S., Prasad P.R. (2010). Synthesis and characterization of 4-hydroxy chalcones using PEG-400 as a recyclable solvent. Res. J. Pharm. Biol. Chem. Sci..

[22] Boukhvalov D.W., Dreyer D.R., Bielawski C.W., Son Y.W. (2012). A computational investigation of the catalytic properties of graphene oxide: Exploring mechanisms by using DFT methods. Chem. Cat. Chem..

[23] Landarani-Isfahani A., Safari J., Ghotbinejad M., Gandomi-Ravandi S., Moshtael (2009). Silica sulfuric acid (SSA), a novel catalyst for synthesis of some-α-phenylhydrazone-2-ketomethylquinolines. Org. Chem. An. Indian J..

[24] Mobinikhaledi A., Foroughifar N., Khodaei H. (2010). Synthesis of octahydroquinazolinone derivatives using silica sulfuric acid as an efficient catalyst. Eur. J. Chem..

[25] Azizian J., Mohammadi A.A., Soleimani E., Karimi A.R., Mohammadizadeh M.R. (2006). A stereoselective three-component reaction: One-pot synthesis of *cis*-isoquinolonic acids catalyzed by silica sulfuric acid under mild and heterogeneous conditions. J. Heterocycl. Chem..

[26] Wu H., Lin W., Wan Y., Xin H.Q., Shi D.Q., Shi Y.H., Yuan R., Bo R.C., Yin W. (2010). Silica gel-catalyzed one-pot synthesis in water and fluoroscene properties studies of 5-amino-2-aryl-3H-chromeno[4,3,2-de][1,8]naphthyridine-4-carbonitriles and 5-amino-2-aryl-3H-quinolino [4,3,2-de][1,6]naphthyridine-4-carbonitriles. J. Comb. Chem..

[27] Cao C., Xu C., Lin W., Li X., Hu M., Wang J., Huang Z., Shi D., Wang Y. (2013). Microwave-assisted improved synthesis of pyrrolo[2,3,4-kl]acridine and dihydropyrrolo[2,3,4-kl]acridine derivatives catalyzed by silica sulfuric acid. Molecules.

[28] Ziarani G.M., Badiei A., Abbasi A., Farahani Z. (2009). Cross-aldol condensation of cycloalkanones and aromatic aldehydes in the presence of nanoporous silica-based sulfonic acid (SiO_2_-Pr-SO_3_H) under solvent free conditions. Chin. J. Chem..

[29] Wang Y., Yuan Y.Q., Guo S.R. (2009). Silica sulfuricacid promotes Aza-Michael addition reactions under solvent-free condition as a heterogeneous and reusable catalyst. Molecules.

[30] Wu H., Shen Y., Fan L.Y., Wan Y., Wang W.X., Shi D.Q. (2006). Solid silica sulfuric acid (SSA) as a novel and efficient catalyst for acetylation of aldehydes and sugars. Tetrahedron.

[31] Kiasat A.R., Kazemi F., Mehrjardi M.F. (2006). Protection of carbonyl groups as 2,4-dinitro-phenyldrazone catalyzed by silica sulfuric acid. Asian J. Chem..

[32] Aoyama T., Kubota S., Takido T., Kodomari M. (2011). Silica sulfuric acid-promoted deacylation of α-bromo-β-diketones. Chem. Lett..

[33] Ghorbani-Choghamarani A., Zamani P. (2011). Ammonium bromide as an effective and viable catalyst in the oxidation of sulfides using nitro urea and silica sulfuric acid. J. Iran. Chem. Soc..

[34] Maleki B., Shirvan H.K., Taimazi F., Akbaradeh E. (2012). Sulfuric acid immobilized on silica gel as highly efficient and heterogeneous catalyst for the one-pot synthesis of 2,4,5-triaryl-1H-imidazoles. Int. J. Org. Chem..

[35] Zolfigol M.A. (2001). Silica sulfuric acid/NaNO_2_ as a novel heterogeneous system for production of thionitrites and disulfides under mild conditions. Tetrahedron.

[B36-molecules-18-10081] 36.Crystallographic data of **5g** have been deposited with the Cambridge Crystallographic Data Centre as supplementary publication No. 950124. These data can be obtained free of charge from The Cambridge Crystallographic Data Centre via www.ccdc.cam.ac.uk/data_request/cif.

[B37-molecules-18-10081] 37.Crystallographic data of **7b** have been deposited with the Cambridge Crystallographic Data Centre as supplementary publication no. 950123. Both X-ray structures were obtained by Professor Muhammad Nawaz Tahir, Department of Physics, University of Sargodha, Pakistan.

[38] Syam S., Abdelwahab S.I., Al-Mamary M.A., Mohan S. (2012). Synthesis of chalcones with anticancer activity. Molecules.

[39] Zheng C.J., Jiang S.M., Chen Z.H., Ye B.J., Piao H.R. (2011). Synthesis and anti-bacterial activity of some heterocyclic chalcone derivatives bearing thiofuran, furan, and quinoline moieties. Arch. Pharm..

[40] Sharma B. (2011). Comparative study of conventional and microwave assisted synthesis of chalcones. Asian J. Chem..

[41] Li J.P., Zhang Y.X., Ji Y. (2008). Selective 1,4-reduction of chalcones with Zn/NH_4_Cl/C_2_H_5_OH/ H_2_O. J. Chin. Chem. Soc..

[42] Camps P., Domingo L.R., Formosa X., Galdeano C., González D., Muñoz-Torrero D., Segalés S., Font-Bardia M., Solans X. (2006). Highly diastereoselective one-pot synthesis of spiro{cyclopenta[*a*]indene-2,2′-indene}diones from 1-indanones and aromatic aldehydes. J. Org. Chem..

[43] (1975). Gazzetta Chimica Italiana.

[44] El-Rayyes N., Al-Qatami S., Edun M. (1987). Heterocycles. 14. Synthesis of 5H-indenopyrimidines. J. Chem. Eng. Data.

[45] Rothenberg G., Downie A.P., Raston C.L., Scott J.L. (2001). Understanding solid/solid organic reactions. J. Am. Chem. Soc..

[46] Kamakshi R., Latha S.S., Reddy B.S.R. (2010). An efficient synthesis of bio-active flourescent benzylidene tetralones. Indian J. Chem..

[47] Sultan A., Raza A.R., Tahir M.N. (2013). Free radical mediated chemoselective reduction of enones. Synth. Commun..

